# Genetic Characterization and Insular Habitat Enveloping of Endangered Leaf-Nosed Bat, *Hipposideros nicobarulae* (Mammalia: Chiroptera) in India: Phylogenetic Inference and Conservation Implication

**DOI:** 10.3390/genes14030765

**Published:** 2023-03-21

**Authors:** Shantanu Kundu, Manokaran Kamalakannan, Tanoy Mukherjee, Dhriti Banerjee, Hyun-Woo Kim

**Affiliations:** 1 Department of Marine Biology, Pukyong National University, Busan 48513, Republic of Korea; 2 Western Ghat Regional Centre, Zoological Survey of India, Kozhikode 673006, India; 3 Agricultural and Ecological Research Unit, Indian Statistical Institute, Kolkata 700108, India; 4 Zoological Survey of India, M Block, New Alipore, Kolkata 700053, India; 5 Marine Integrated Biomedical Technology Center, National Key Research Institutes in Universities, Pukyong National University, Busan 48513, Republic of Korea

**Keywords:** Chiroptera, Hipposideridae, systematics, phylogeny, endemic species, conservation

## Abstract

**Simple Summary:**

The present study provides the first genetic data on *Hipposideros nicobarulae*, an endangered and endemic leaf-nosed bat in the Nicobar Islands, India. Preliminary data sheds light on the phylogenetic relationship of *H. nicobarulae* and other closely related species (*H.* cf. *antricola* and *H.* cf. *einnaythu*) from South and Southeast Asia. Additionally, the distribution modelling provides information on suitable habitats for *H. nicobarulae* in the insular biogeography of Nicobar Island. Further integrated studies are needed to clarify the evolution, population genetics, distribution, and diversification of the *Hipposideros* species in the world.

**Abstract:**

The Nicobar leaf-nosed Bat (*Hipposideros nicobarulae*) was described in the early 20th century; however, its systematic classification has been debated for over 100 years. This endangered and endemic species has achieved species status through morphological data in the last 10 years. However, the genetic information and phylogenetic relationships of *H. nicobarulae* remain neglected. The generated mitochondrial cytochrome b gene (mtCytb) sequences (438 bp) of *H. nicobarulae* contains 53.42–53.65% AT composition and 1.82% variable sites. The studied species, *H. nicobarulae* maintains an 8.1% to 22.6% genetic distance from other *Hipposideros* species. The genetic divergence estimated in this study is congruent with the concept of gene speciation in bats. The Bayesian and Maximum-Likelihood phylogenies clearly discriminated all *Hipposideros* species and showed a sister relationship between *H. nicobarulae* and *H.* cf. *antricola*. Current mtCytb-based investigations of *H. nicobarulae* have confirmed the species status at the molecular level. Further, the MaxEnt-based species distribution modelling illustrates the most suitable habitat of *H. nicobarulae* (294 km^2^), of which the majority (171 km^2^) is located on Great Nicobar Island. The present study suggests rigorous sampling across the range, taxonomic coverage, the generation of multiple molecular markers (mitochondrial and nuclear), as well as more ecological information, which will help in understanding population genetic structure, habitat suitability, and the implementation of appropriate conservation action plans for *H. nicobarulae* and other *Hipposideros* species.

## 1. Introduction

Bats (order Chiroptera) are the second most diverse group of mammals after Rodentia and currently contain approximately 1456 species worldwide [1]. Among them, more than 130 bat species under nine families have been reported from the Indian mainland and islands. The Palaeotropical genus *Hipposideros* consists of about 74 species worldwide, and 12 species are distributed in the Indian subcontinent [2]. Among them, four species (*Hipposideros diadema, Hipposideros fulvus*, *Hipposideros grandis*, and *Hipposideros larvatus*) are distributed in the Andaman group of islands, whereas three species (*H. diadema, Hipposideros gentilis,* and *Hipposideros nicobarulae*) are reported from the Nicobar group of islands, India [3,4,5,6,7]. To date, *H. nicobarulae* is only known from the type locality, Nicobar Island in the Andaman and Nicobar archipelago in India [3,4]. The Nicobar Leaf-nosed Bat, *H. nicobarulae*, was originally described in the early 20th century [8], and for over 100 years this bat species (or “this form”) was considered a subspecies of *Hippisideros ater* as *H. ater nicobarulae* [9,10]. However, based on its body size and external and dental morphology, this bat species (or ‘this form’) was elevated to a distinct species in the recent past [3]. Due to the lack of relevant material, the species status of *H. nicobarulae* is still controversial and has not yet been tested using genetic data. *Hippisideros nicobarulae* lives in a mixed-sex colony in caves, abandoned buildings, and tunnels as well [3,4]. Due to habitat destruction by tsunamis and other anthropogenic activities, the population size of *H. nicobarulae* is decreasing day by day [4]. Hence, the species is classified as “endangered” by the Bat Specialist Group of the IUCN Red List of Threatened Species [11].

With the rapid advancement of molecular tools, integrated methods incorporating partial mitochondrial and nuclear genes, complete genomes, and transcriptome data were used in an iterative manner to identify and elucidate the diversity of bats worldwide [12,13,14,15]. A number of molecular approaches have been published in *Hipposideros* bats and related taxa in various contexts: description of new species or subspecies [16], detection of cryptic species [17,18], phylogenetic and evolutionary significance [19,20], and phylogeographic studies [21,22]. Additionally, being an indicator species, many physiological studies on bats have also been accomplished around the world [23,24]. The assessment of the mitochondrial Cytochrome b (mtCytb) gene has proven useful for identification and genetic diversity estimation in mammals, including bats [25,26,27]. Nevertheless, the genetic information of *H. nicobarulae* is still not available to the scientific community. In this milieu, we examined bat specimens collected from the Nicobar Islands through an integrated approach to improve our understanding of the systematic position of this charismatic taxon. This preliminary data will enrich the global genetic library and encourage further phylogeographic research on endangered *H. nicobarulae* and other closely related species.

Additionally, apart from existing threats to *H. nicobarulae*, the insular island habitat automatically increases the vulnerability of this species. To protect any threatened species, it is important to know how different taxa respond to land use change and how habitat and climate change affect their persistence [28,29]. Several studies have already been conducted to achieve habitat distribution modelling of bats globally [30,31,32] and in India [33]. Due to the lack of sufficient knowledge on habitat fitness, the present study also attempts to perform species distribution modelling of *H. nicobarulae* to identify a suitable home range in the Nicobar Islands, which can be used for monitoring and strategic conservation of this species in the near future.

## 2. Materials and Methods

### 2.1. Ethics Statement and Sampling

**Figure 1:**
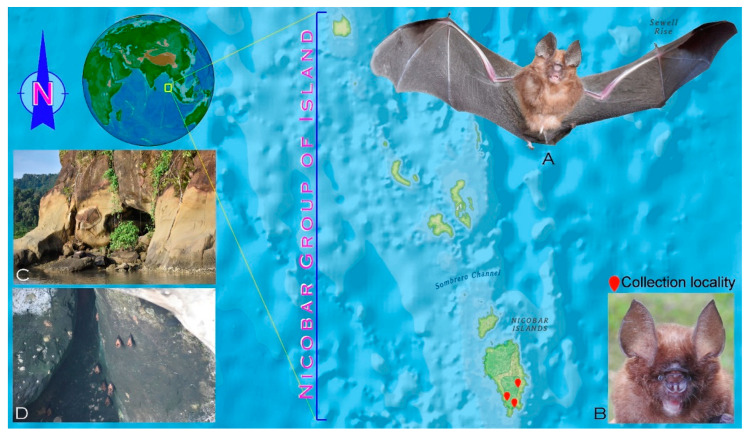
Map showing the collection localities of *H. nicobarulae* in the Great Nicobar Island (marked by a red pin). (**A**) A live specimen of *H. nicobarulae* captured by M. Kamalakannan during the field survey in the Andaman and Nicobar archipelago; (**B**) a frontal view of the ears and nose leaves of *H. nicobarulae*; (**C**,**D**) the habitat of *H. nicobarulae* on the Great Nicobar Island, India.

A total of six individuals (four males and two females) of *H. nicobarulae* were collected from three different localities at Great Nicobar Island (6.83 N 93.86 E, 6.85 N 93.81 E, and 6.94 N 93.87 E) by using a mist net (Figure 1). The morphological examination was carried out at the Western Ghat Regional Centre (WGRC) of the Zoological Survey of India (ZSI), Kerala, India. The species was identified by the morphological characters described earlier [3]. A photograph of the live specimen was taken by M. Kamalakannan (Figure 1). The specimens were vouchered in 10% formaldehyde for long-term preservation. The voucher specimens were deposited in the National Zoological Collections of the WGRC, ZSI, under the museum catalogue numbers (ZSIWGRC-3630 to ZSIWGRC-3635). A meager amount of tissue sample was collected from the toe region of each sample for molecular experiments. The experimental procedures were approved by the Zoological Survey of India. No specific permission was required for sampling the targeted taxa.

### 2.2. Morphological Identification

The external morphology and cranial characteristics were measured for each sample of *H. nicobarulae* by using vernier calipers to the nearest 0.01 mm. To explain the species level identity and distinct specific status, the morphometrics data of closely related (*H. ater* and *H.* cf. *einnaythu*) and sympatric (*H. diadema*, *H. fulvus*, *H. grandis*, *H. larvatus*, and *H. gentilis*) species were acquired from the previous literature and compared [3,34,35,36] (Table 1). We could not obtain the taxonomic measurements of *H.* cf. *antricola* from any sources; hence, not incorporated here. The external measurements were performed according to the Bates and Harrison, 1997 [34]; length of head and body (HBL): from the tip of the snout to base of the tail on dorsal side, length of forearm (FA): from the extremity of the elbow to the extremity of the carpus with the wings folded, ear length (EL): from the lower border of the externally auditory meatus to the tip of the pinna, length of hindfoot (HF): from the extremity of the heel behind the Os calcis to the extremity of the longest digit, length of tibia (TIB): from the knee joint to the ankle, length of tail (TL): from the tip of the tail to its base adjacent to the body.

### 2.3. Mitochondrial DNA Extraction and Sequencing

The present study targeted the amplification of only a small fragment (>400 bp) of the mitochondrial Cytb gene, as it has been shown to be suitable for mammalian species identification [27,37]. Total genomic DNA was extracted by the standard phenol-chloroform isoamyl alcohol method and visualized in 1% agarose gel electrophoresis [38]. To amplify the partial mtCytb gene, mcb 398: 5′-TACCATGAGGACAAATATCATTCTG-3′ and mcb 869: 5′-CCTCCTAGTTTGTTAGGGATTGATCG-3′) primer pairs were used [37]. The 30 mL PCR mixture contains 10 pmol of forward and reverse primer, 20 ng of template DNA, 1X PCR buffer, 1.0–1.5 mM of MgCl_2_, 0.25 mM of each dNTPs, and 1U of Taq DNA polymerase (Invitrogen). The PCR was executed in a Veriti Thermal Cycler (Applied Biosystems) with the standard thermal settings. The PCR products were purified by using a QIAquick Gel Extraction Kit (QIAGEN). The cycle sequencing was performed using the BigDye Terminator ver. 3.1 Cycle Sequencing Kit (ABI) and 3.2 pmol of each primer. The PCR products were purified by the BigDye X-terminator kit (ABI) and bidirectionally sequenced by the genetic analyser housed at the Rajiv Gandhi Centre for Biotechnology (RGCB), Thiruvananthapuram, Kerala, India.

### 2.4. Sequence Annotation and Dataset Construction

To avoid the pseudogenes in the raw sequences, the noisy parts of each chromatogram were brought down at both ends through the SeqScanner Version 1.0 (Applied Biosystems). To validate the generated sequence, both nucleotide BLAST (https://blast.ncbi.nlm.nih.gov, accessed on 10 March 2023) and ORF finder (https://www.ncbi.nlm.nih.gov/orffinder/, accessed on 10 March 2023) were used to check the insertion-deletion and amino acid array for the vertebrate mitochondrial gene. The annotated sequences were submitted to GenBank’s global database through Bankit (https://www.ncbi.nlm.nih.gov/WebSub/, accessed on 10 March 2023). To estimate the genetic distances and perform the phylogenetic analysis, a total of 98 mtCytb sequences of *Hipposideros* species were acquired from NCBI GenBank. The mtCytb sequence of *Rhinolophus rouxii* (family Rhinolophidae) (accession no. JQ316214) was used as an out-group in the present phylogenetic analyses. The generated and database sequences were aligned through ClustalX software to build the final dataset [39].

### 2.5. Genetic Divergence and Phylogenetic Analyses

The Kimura-2-parameter (K2P) genetic divergence was estimated by MEGAX [40]. The best-fit model was estimated through JModelTest v2 with the lowest BIC (Bayesian Information Criterion) value [41]. The Bayesian (BA) topology was constructed in Mr. Bayes 3.1.2 by choosing nst = six for the GTR + G + I with one cold and three hot chains of metropolis-coupled Markov Chain Monte Carlo (MCMC); it was run for 10,000,000 generations with 25% burn-in and trees saving at every 100 generations [42]. The maximum-likelihood (ML) tree was constructed through the IQ-Tree web server with the GTR + G + I model and 1000 bootstrap samples [43]. The final topologies were illustrated in the web-based iTOL tool (https://itol.embl.de/, accessed on 10 March 2023) and edited with Adobe Photoshop CS 8.0 [44].

### 2.6. Species Occurrence Data

The occurrence records of *H. nicobarulae* were collected from available literature [3,4], and the GBIF online data repository (https://doi.org/10.15468/dl.d8ph4a, accessed on 10 March 2023) [45]. We started with a set of 21 habitat variables grouped into three types: climatic, land cover and land use (LULC), and topographic (Table S1). The climatic variables were represented by 19 bioclimatic variables from Worldclim, Ver: 2.0 (https://www.worldclim.org/, accessed on 10 March 2023) [46]. To examine the influence of individual LULC classes, land use and land cover derived from Copernicus Global Land Service (https://lcviewer.vito.be/download, accessed on 10 March 2023) were used. The elevation profile for the study landscape was generated using the 90-m Shuttle Radar Topography Mission (SRTM) data (http://srtm.csi.cgiar.org/srtmdata/, accessed on 10 March 2023). Finally, all predictors were resampled at 30 arc sec spatial resolution, and spatial multicollinearity among the variables were eliminated by using Pearson’s correlation with r > 0.8 in SDM Toolbox v2.4. 

### 2.7. Model Building and Evaluation

For identifying suitable habitats for *H. nicobarulae* in the study landscape, we have attempted maximum entropy-based modelling using the software MaxEnt Ver. 3.4.4. The adopted method provides the probability of occurrence of a given species, ranked from 0 (least likely occurrence) to 1 (most likely occurrence). Our result generates a distribution probability as a probability raster surface ranging from a 0 to 1 value, where 0 is the most unsuitable zone and 1 is the most suitable zone. The relative influence of the selected variables was estimated by the Jackknife test of developed regularized training gain [47]. For model evaluation, the area under the curve statistics (AUC) of the receiver operating characteristic (ROC) curves with values ranging from 0 to 1, where a score of less than 0.5 is worse than random, followed by a score of 0.5 as considered a random prediction. A score between 0.7 and 0.8 is regarded as an acceptable model result, followed by a score between 0.8 and 0.9 as excellent, and <0.9 is regarded as an exceptional model [48]. Finally, we have prepared the binary maps from the continuous suitability raster based on an equal test sensitivity and specificity (SES) threshold.

## 3. Results 

### 3.1. Taxonomic Identification 

**Figure 2:**
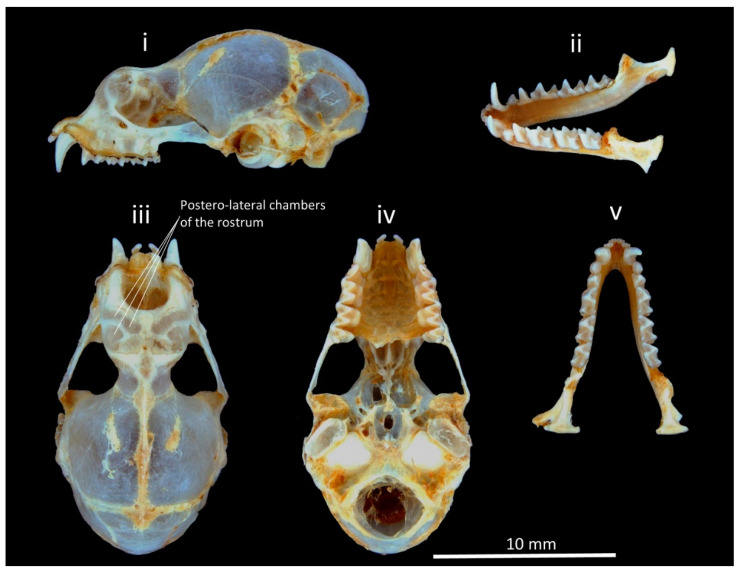
Craniodental characters (skull and mandible) of *H. nicobarulae*: (**i**) lateral view of maxilla; (**ii**) lateral-ventral view of mandible; (**iii**) dorsal view of maxilla; (**iv**) ventral view of maxilla; and (**v**) occlusal view of mandible.

The collected specimens were identified as *H. nicobarulae* by the external morphometric characteristics: head and body length (HBL) ranges 41.6–47.9 mm; forearm length (FA): 38.1–42.2 mm; ear length 15.50–15.94 mm; hindfoot length: 7.67–8.05 mm; tibia length: 17.23–18.44 mm; tail length: 24.7–32.5 mm; and craniodental measurements: greatest length of the skull (GTL) ranges 16.68–17.23 mm: condylobasal length (CBL): 16.05–16.38 mm; condylocanine length (CCL): 15.07–15.42 mm; zygomatic breadth (ZB): 8.28–8.62 mm; breadth of the braincase (BB): 7.26–7.50 mm; maxillary toothrow length (C–M^3^): 5.69–5.74 mm; mandibular toothrow length (C–M_3_): 6.00–6.29 mm; posterior palatal width (M^3^–M^3^): 4.22–4.29 mm; anterior palatal width (C^1^–C^1^): 3.39–3.53 mm; mandibular length (M): 10.05–10.13 mm. The external and cranial measurements are comparatively larger than the *H. ater* (HBL: 38–48 mm; FA: 34–38 mm; GTL: 15.4–16.5 mm; CBL: 13.6–14.6 mm; CCL: 13.2–14.2 mm; M: 9.2–10.2 mm); the ears are large; the nose-leaf is slightly elongated and narrowed; there are no supplementary lateral leaflets; and the postero-lateral chambers of the rostrum are inflated but are significantly smaller than the *H. ater* (Figure 1 and Figure 2). The external morphological measurements of other closely related *Hipposideros* species, namely, *H. einnaythu, H. fulvus, H. grandis, H. larvatus,* and *H. gentilis,* overlap with *H. nicobarulae* except for *H. diadema, H. grandis, H. gentilis,* and *H. larvatus,* where these four species are significantly larger than *H. nicobarulae* (Table 1).

### 3.2. Molecular Identification 

**Figure 3:**
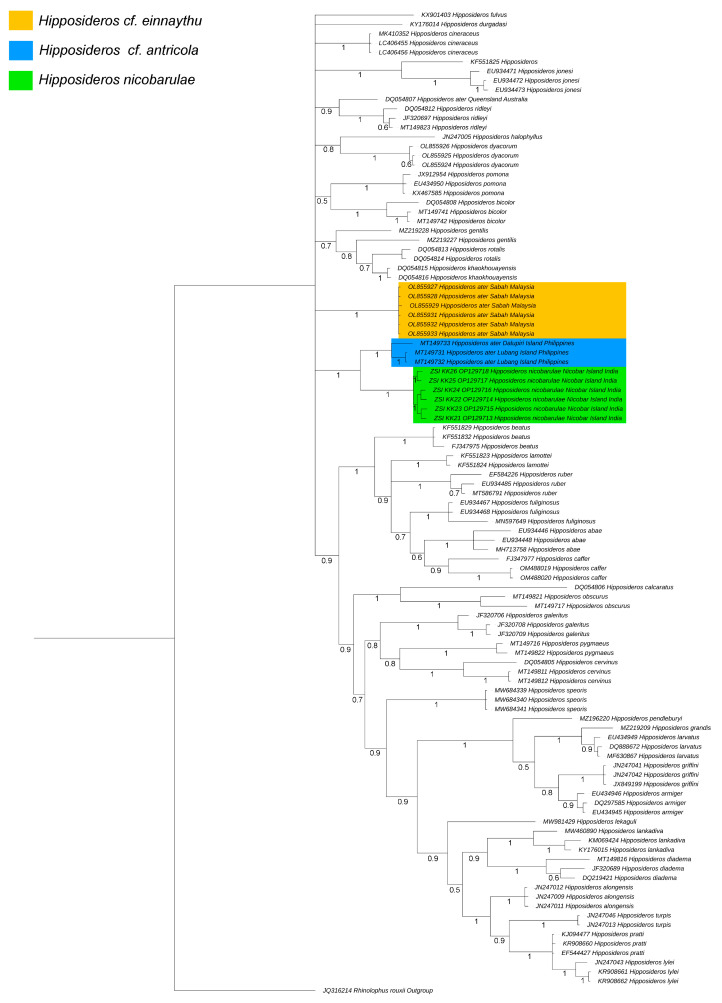
The BA phylogeny based on the mtCytb gene clearly discriminates all *Hipposideros* species in this present study. The Genebank accession number is indicated in parentheses along with the species name. The posterior probability support values are mentioned with each node. The relationship between *H. nicobarulae* and closely related species is mentioned in different colors.

The partial cytochrome b analyses included 104 mtCytb sequences from 40 putative species. The generated sequences revealed 92.41% to 92.63% similarity with *H. ater* sequences (MT149731-MT149733) generated from the Philippines in the global nucleotide BLAST search. The mtCytb sequence (438 bp) contains an average of 53.55% AT composition in all *Hipposideros* species, with 53.42–53.65% in *H. nicobarulae*. A total of 49.54% variable sites were observed in the partial mtCytb sequences among all *Hipposideros* bats, while 1.82% variable sites were found in *H. nicobarulae*. The present dataset showed a 15.9% overall K2P mean genetic distance in all *Hipposideros* species. The interspecies genetic distances ranged from 4.1% (between *H. grandis* and *H. larvatus*) to 31% (*H. grandis* and *H. fulvus*). The studied species, *H. nicobarulae* maintains 8.1% to 22.6% genetic distance from other species in the present dataset (Table S2). Due to a lack of mtCytb data, genetic comparisons of *H. nicobarulae* with *H. ater* distributed in mainland India and Sri Lanka were not possible in this present analysis. However, *H. nicobarulae* showed 8.1% distance with *H. ater* (*H.* cf. *antricola*) generated from the Philippines, 13.5% distance with *H. ater* (*H.* cf. *einnaythu*) generated from Malaysia, and 12.4% distance with *H. ater* (hitherto unreported species) from Queensland, Australia. Besides, *H. nicobarulae* also maintained high genetic distances with other sympatric species (11.9% with *H. gentilis*, 16.8% with *H. diadema*, 18.2% with *H. larvatus*, 19.3% with *H. grandis*, and 22.6% with *H. fulvus*) distributed in the Andaman and Nicobar Islands (Table S2). In BA phylogeny, all *Hipposideros* species were clustered together (Figure 3). The ML tree also showed similar topology of the studied *Hipposideros* species (Figure S1). The studied species, *H. nicobarulae,* cohesively cladded with *H.* cf. *antricola* (distributed in the Philippines) and was recovered as a sister species. Further, the *H.* cf. *einnaythu* (distributed in Malaysia) showed close clustering with the *H. nicobarulae + H.* cf. *antricola* clades in the present BA phylogeny (Figure 3). As anticipated, the *Hipposideros* species were clearly discriminated by this fast-evolving mtCytb gene. We knew that the short sequence of a single mitochondrial gene does not clearly affect the understanding of phylogenetic relationships, but we were able to confirm the species-level genetic information of this endangered bat species, which will help for future phylogenetic, evolutionary, and population genetic studies.

### 3.3. Model Performance and Habitat Suitability

**Figure 4:**
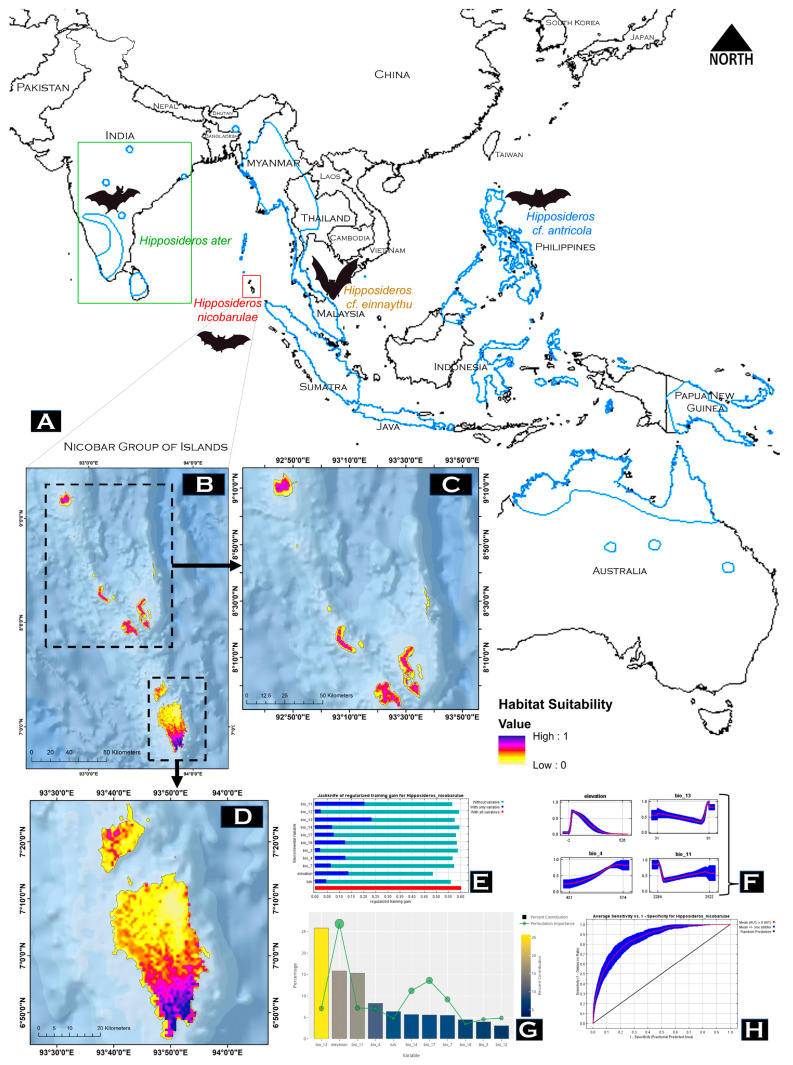
(**A**) The distribution pattern of *H. nicobarulae* and other closely related species (*H.* cf. *einnaythu*, and *H.* cf. *antricola*), elevated as a distinct species from *H. ater*, (**B**) Representing the probability of suitable habitats of *H. nicobarulae* in its extent distribution range, (**C**) Representing the distribution probability in the northern range within Car Nicobar, Terresa, Kamorta, Katchal and Nancowry Islands, (**D**) Representing the distribution probability in the southern range within Little Nicobar and Great Nicobar. (**E**) Jackknife test for all the eleven variables (**F**) Response curves of the important variables for habitat suitability of *H. nicobarulae*, (**G**) Percentage contribution is represented by a column graph (color ramp = percentage contribution), and permutation importance is represented by the circular plot (size = permutation importance). (**H**) The average training ROC for the final model replicates.

Our result has precisely predicted the suitable habitats within its extent distribution range. The average training AUC score for replicate runs (*n* = 50) for the model was found to be 0.861 ± 0.021 (SD) (Figure 4). Out of the total distribution range extent (1841 km^2^), only 294 km^2^ (15.96%) was found suitable for *H. nicobarulae*. The most suitable areas are within the southern range of Grater Nicobar (171 km^2^). Further, within the smaller islands, the most suitable and continuous habitat patches (40 km^2^) were distributed in the Car Nicobar. The result suggests that the suitable distribution for *H. nicobarulae* was greatly influenced by the precipitation of the wettest month, with a relative contribution of 25.80%, followed by the contribution of elevation at 15.80% (Figure 4). Further, the distribution probability concerning the elevation was found within the range of 0–300 m, after which the probability values were found to reduce substantially. 

## 4. Discussion

The *Hipposideros* bats are distributed in the Palearctic, Afrotropical, Indo-Malayan, and Australasian regions and certainly contain many unresolved systematic issues. Some of them concern the taxonomic position of certain species that were not previously included in the relevant studies for various reasons (rareness in collections and in nature, limited range in areas inaccessible to researchers, etc.). Clarification of the taxonomic position is especially important for narrow-range endemics since it affects the development and implementation of conservation measures. Thus, taking into account the uncertainties with the taxonomy of *H. nicobarulae*, the present study elucidates an interesting systematic doubt both morphologically and genetically, as well as in terms of their habitat suitability. 

The Great Nicobar Island is located about 170 km northwest of Sumatra, 350 km southeast of the Little Andaman Islands, 640 km off the southwest coast of Thailand, and 1306 km off the east coast of Sri Lanka. These islands are evidenced to be a discrete biogeographic unit and hold many magnetic faunal components, which act as a significant model for evolutionary studies [49]. During the Miocene–Pliocene, volcanic eruptions formed many isolated islands, and their sporadic connectivity was directly linked to changes in sea level during the Pleistocene, which accorded both geographical and temporal processes of species diversification in South and Southeast Asia [50]. The bathymetric study unwrapped the invisible connectivity of the Andaman and Nicobar archipelago and Sumatra by a well-developed seamount on the seafloor [51]. Moreover, the unparalleled biogeography of Southeast Asian oceanic islands provides a suitable habitat for many *Hipposideros* bats [52,53]. Looking at the biogeographic pattern, it is obvious that the species diversity of the Andaman and Nicobar archipelago would be more alike to the Indo-Malayan and Sundaic realms. 

Considering the molecular-based species delimitation performed in the present mtCytb dataset, *H. nicobarulae* is genetically distinct and evolutionary close to its postulated sister species, *H.* cf. *antricola,* compared with other mainland species (*Hipposideros pomona* and *Hipposideros durgadasi*) of India. Previous studies have suggested that ≥8% genetic distance in the mitochondrial gene is pretty significant for inferring the genetic species concept in bats [26,54]. Similar genetic diversity has also been identified in Neotropical and Palearctic bats [55,56]. The targeted taxa (*H. nicobarulae*) maintain high genetic divergence (8.1% to 22.6%) with other *Hipposideros* bats, congruent with previous bat genetic speciation concepts. Further, the paraphyletic cladding of *Hipposideros* taxa from the African and Asian continents eliminates the biogeographical paradox and provides an interesting example of convergent evolution [57,58]. Moreover, the present integrated approach confirms the evolutionary placement of *H. nicobarulae* and is congruent with the prevailing hypothesis of *Hipposideros* classification. This cladding pattern and unique biogeography indicate that the endemic *H. nicobarulae* is not the result of local radiation, as might initially be assumed by *H. ater* sensu lato, but appears to have arisen from multiple independent colonization events. However, a more comprehensive study is required, including the generation of more molecular data (mitochondrial and nuclear) supporting many of the deeper nodes of phylogeny and their echolocation divergence, to understand their astonishing evolutionary pattern and diversification that may shed light on the speciation mechanisms of *Hipposideros* bats in their range distribution. 

The mammalian fauna of the Great Nicobar Island comprises mostly bats and a few non-volant mammals. A total of 25 species of bats under 13 genera are found in the Andaman and Nicobar archipelago [4,34]. Among them, seven species under six genera were reported from the Great Nicobar Island (*Cynopterus brachyotis*, *Hipposideros diadema*, *H. gentilis*, *H. nicobarulae*, *Myotis horsfieldii*, *Murina cyclotis*, *Pteropus melanotus*, and *Taphozous melanopogon*). Apart from *H. nicobarulae* (endangered) and *P. melanotus* (vulnerable), other species are “least concern” in this insular habitat. It is proven that climate change and consequent natural calamities like tsunamis and earthquakes are gradually increasing the sea level of the Indian Ocean, which is greatly affecting the ecosystems of the Andaman and Nicobar Islands [59,60]. It is therefore imperative to select and conserve a minimum land area for biodiversity conservation that will also help conserve endemic and threatened species within an ecosystem [61,62]. Therefore, the conservation of the estimated suitable habitat (294 km^2^) is crucial for the conservation of the endemic *H. nicobarulae* population within this isolated insular habitat. Our predictions about habitat suitability will help governments and conservation organizations when formulating conservation policies for these endangered species. We also recommend further ecology studies of this species within its total range (1841 km^2^) to understand its present status and implement upcoming action plans for proper conservation.

## 5. Conclusions

The population of *H. nicobarulae* has declined significantly on Nicobar Island due to climate change and habitat degradation. No proper conservation action has been aimed to protect this species as its taxonomic status is still controversial in the scientific community. Although morphological data overlapped with some *Hipposideros* species, genetic data clearly discriminated most of the species and showed a matrilineal relationship with *H. nicobarulae* and *H.* cf. *antricola* in the present dataset. The study also delineated the suitable habitats and conservation priority areas of *H. nicobarulae* on Nicobar Island. We recommend an integrated approach, prioritizing extensive sampling, taxonomic coverage, multiple loci enrichment, and ecological modelling, to gain evolutionary knowledge and conserve this threatened bat species in the wild. 

## Figures and Tables

**Table 1:** External morphological measurements (mm) of *Hipposideros* spp. [3,34,35,36]. The measurements are represented in range (minimum and maximum). HBL: head and body length; FA: forearm length; EL: ear length; HF: hind foot length; Tib: tibia length; T: tail length.

Species	HBL	FA	EL	HF	Tib	T
*H. nicobarulae* (*n* = 6)	41.6–47.9	38.1–42.2	15.5–15.9	7.67–8.05	17.2–18.4	24.7–32.5
*H. ater* (*n* = 11)	38.0–48.0	34.0–38.0	14.8–20.0	5.30–7.20	15.2–17.8	20.0–30.0
*H. einnaythu* (*n* = 2)	43.3, 49.1	39.5, 40.3	16.6, 16.7	6.5, 7.0	16.2, 17.8	24.7, 28.7
*H. diadema* (*n* = 6)	-	80.2–90.2	23.3–30.8	13.67–18.1	29.7–37.8	44.3–57.2
*H. fulvus* (*n* = 35)	40.0–50.0	38.4–44.0	19.0–26.0	6.0–9.8	16.5–20.7	24.0–35.0
*H. grandis* (*n* = 12)	70.0–79.0	60.0–64.0	21.5–24.0	12.0–13.5	21.0–25.7	34.0–39.0
*H. larvatus* (*n* = 8)	-	48.7–59.0	14.4–23.5	3.9–6.6	19.0–20.1	24.8–35.5
*H. gentilis* (*n* = 9)	36.0–52.0	39.5–43.2	22.0–25.0	6.3–8.5	18.2–19.1	28.0–35.0

## Data Availability

The nucleotide sequence data that support the findings of this study are openly available in GenBank of NCBI at [https://www.ncbi.nlm.nih.gov, accessed on 10 March 2023] under the accession no. OP129713-OP129718.

## Supplementary Materials

The following supporting information can be downloaded at: https://www.mdpi.com/article/10.3390/genes14030765/s1, Figure S1. The mtCytb based ML phylogeny of the studied *Hipposideros* species; Table S1. Primary environmental and topographical variables used for ensemble modelling; Table S2. The K2P genetic divergences of the analysed of the studied *Hipposideros* species.
