# Supplementary material for: Genetic Characterization and Insular Habitat Enveloping of Endangered Leaf-Nosed Bat, Hipposideros nicobarulae (Mammalia: Chiroptera) in India: Phylogenetic Inference and Conservation Implication
Source: Genes (Basel). 2023 Mar 21;14(3):765. doi: 10.3390/genes14030765 (PMC10048616; doi:10.3390/genes14030765)
Supplement: Supplementary file 1 [file genes-14-00765-s001.zip › Table S1.pdf]

Table S1. Primary environmental and topographical variables used for ensemble modelling.

Sl. No.	Code	Variables description
	**Bioclimatic**	
1.	bio_1	Annual Mean Temperature
2.	bio_2	Mean Diurnal Range (Mean of monthly (max temp - min temp)
3.	bio_3	Isothermality (BIO2/BIO7) (* 100)
4.	bio_4	Temperature Seasonality (standard deviation *100)
5.	bio_5	Max Temperature of Warmest Month
6.	bio_6	Min Temperature of Coldest Month
7.	bio_7	Temperature Annual Range (BIO5-BIO6)
8.	bio_8	Mean Temperature of Wettest Quarter
9.	bio_9	Mean Temperature of Driest Quarter
10.	bio_10	Mean Temperature of Warmest Quarter
11.	bio_11	Mean Temperature of Coldest Quarter
12.	bio_12	Annual Precipitation
13.	bio_13	Precipitation of Wettest Month
14.	bio_14	Precipitation of Driest Month
15.	bio_15	Precipitation Seasonality (Coefficient of Variation)
16.	bio_16	Precipitation of Wettest Quarter
17.	bio_17	Precipitation of Driest Quarter
18.	bio_18	Precipitation of Warmest Quarter
19.	bio_19	Precipitation of Coldest Quarter
	**Topographic**	
20.	elevation	Elevation
	**Anthropogenic**	
	**LULC**	
21.	LULC (Categorical)	• Herbaceous Vegetation<br>• Water Bodies<br>• Cropland<br>• Bare land<br>• Shrubland<br>• Forest<br>• Urban
