# Supplementary figures and images for: Genetic Characterization and Insular Habitat Enveloping of Endangered Leaf-Nosed Bat, Hipposideros nicobarulae (Mammalia: Chiroptera) in India: Phylogenetic Inference and Conservation Implication
Source: Genes (Basel). 2023 Mar 21;14(3):765. doi: 10.3390/genes14030765 (PMC10048616; doi:10.3390/genes14030765)

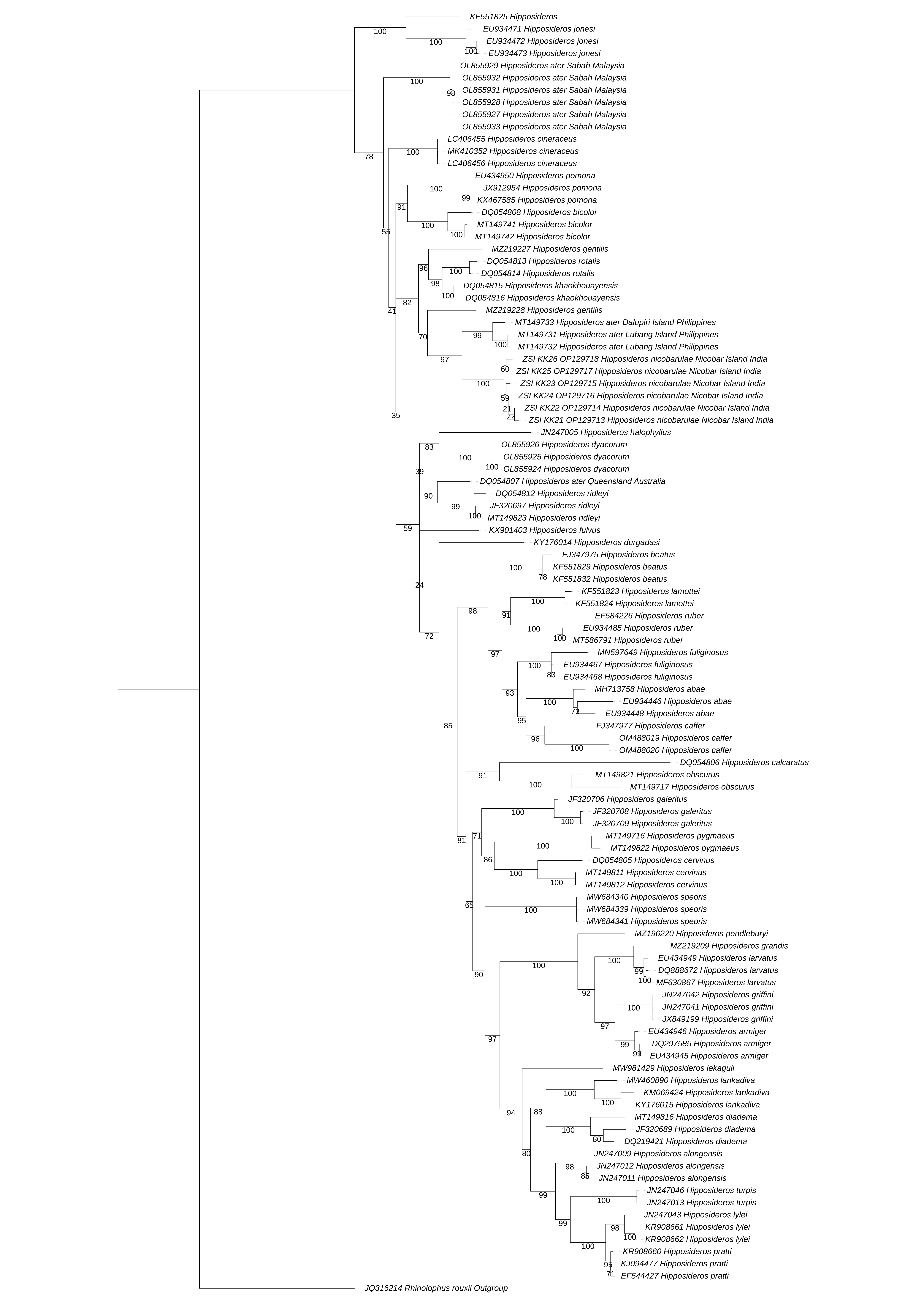

Supplement: Supplementary file 1 [file genes-14-00765-s001.zip › Figure S1.jpg]
